# Gut microbiome and metabolome characteristics of patients with cholesterol gallstones suggest the preventive potential of prebiotics

**DOI:** 10.1002/imt2.70000

**Published:** 2025-02-21

**Authors:** Ye Liu, Hexin Li, Tianhan Sun, Gaoyuan Sun, Boyue Jiang, Meilan Liu, Qing Wang, Tong Li, Jianfu Cao, Li Zhao, Fei Xiao, Fangqing Zhao, Hongyuan Cui

**Affiliations:** ^1^ Peking University Fifth School of Clinical Medicine Beijing Hospital, National Center of Gerontology Beijing China; ^2^ Clinical Biobank, Beijing Hospital, National Center of Gerontology, Institute of Geriatric Medicine, Chinese Academy of Medical Sciences Beijing China; ^3^ The Key Laboratory of Geriatrics, Beijing Institute of Geriatrics, Beijing Hospital, National Center of Gerontology, National Health Commission, Institute of Geriatric Medicine, Chinese Academy of Medical Sciences Beijing China; ^4^ Department of General Surgery, Beijing Hospital, National Center of Gerontology, Institute of Geriatric Medicine Chinese Academy of Medical Sciences & Peking Union Medical College Beijing China; ^5^ Department of Gastroenterology, Beijing Hospital, National Center of Gerontology, Institute of Geriatric Medicine Chinese Academy of Medical Sciences Beijing China; ^6^ Institute of Zoology, Chinese Academy of Sciences Beijing China; ^7^ Key Laboratory of Systems Biology, Hangzhou Institute for Advanced Study, Beijing Institutes of Life Science Chinese Academy of Sciences Beijing China

**Keywords:** cholesterol gallstone, gut microbiota, metabolomics, microbiome‐targeted therapies, transplantation

## Abstract

Cholesterol gallstones (CGS) still lack effective noninvasive treatment. The etiology of experimentally proven cholesterol stones remains underexplored. This cross‐sectional study aims to comprehensively evaluate potential biomarkers in patients with gallstones and assess the effects of microbiome‐targeted interventions in mice. Microbiome taxonomic profiling was conducted on 191 samples via V3−V4 16S rRNA sequencing. Next, 60 samples (30 age‐ and sex‐matched CGS patients and 30 controls) were selected for metagenomic sequencing and fecal metabolite profiling via liquid chromatography‐mass spectrometry. Microbiome and metabolite characterizations were performed to identify potential biomarkers for CGS. Eight‐week‐old male C57BL/6J mice were given a lithogenic diet for 8 weeks to promote gallstone development. The causal relationship was examined through monocolonization in antibiotics‐treated mice. The effects of short‐chain fatty acids such as sodium butyrate, sodium acetate (NaA), sodium propionate, and fructooligosaccharides (FOS) on lithogenic diet‐induced gallstones were investigated in mice. Gut microbiota and metabolites exhibited distinct characteristics, and selected biomarkers demonstrated good diagnostic performance in distinguishing CGS patients from healthy controls. Multi‐omics data indicated associations between CGS and pathways involving butanoate and propanoate metabolism, fatty acid biosynthesis and degradation pathways, taurine and hypotaurine metabolism, and glyoxylate and dicarboxylate metabolism. The incidence of gallstones was significantly higher in the *Clostridium glycyrrhizinilyticum* group compared to the control group in mice. The grade of experimental gallstones in control mice was significantly higher than in mice treated with NaA and FOS. FOS could completely inhibit the formation of gallstones in mice. This study characterized gut microbiome and metabolome alterations in CGS. *C. glycyrrhizinilyticum* contributed to gallstone formation in mice. Supplementing with FOS could serve as a potential approach for managing CGS by altering the composition and functionality of gut microbiota.

## Highlights

- Metabolites of amino acids, fatty acids, and nicotinic acid may affect the synthesis, transport, or metabolism of cholesterol, thereby impacting gallstone formation.
- Oral administration of *C. glycyrrhizinilyticum* can contribute to cholesterol gallstone formation.
- Fructooligosaccharides could serve as an inexpensive microbiota‐targeted therapeutic option for cholesterol gallstones.

## Graphical abstract

Alterations in gut microbial composition and fecal metabolome have been reported in patients with gallstones. The gut microbiota plays a crucial role in shaping the host's fecal metabolic profile. Specifically, metabolites of amino acids, fatty acids, and nicotinic acid may affect the synthesis, transport, or metabolism of cholesterol, thereby impacting gallstone formation. Oral administration of *C. glycyrrhizinilyticum* can promote histological liver damage and affect liver lipid metabolism, contributing to cholesterol gallstone formation. Fructooligosaccharides can be a new, cost‐effective, and relatively simple microbiota‐targeted therapeutic approach for cholesterol gallstones. Microbiome‐targeted therapies, involving the modulation of gut microbiota, should be explored as potential treatments for gallstones.

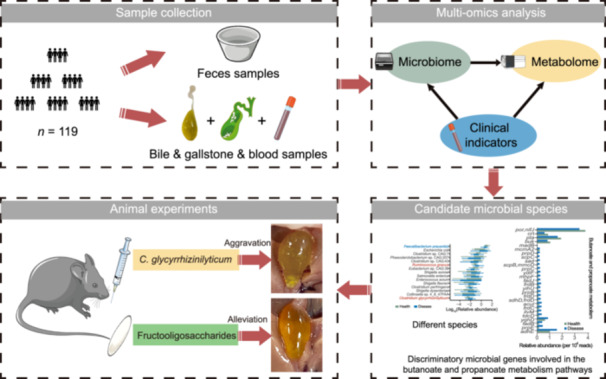

## INTRODUCTION

Gallstones are a common disease that occurs frequently. Gallstones affect around 10%–15% of adults, leading to significant discomfort [1]. 85%–90% of gallstones are cholesterol gallstones (CGS). Both environmental and genetic factors contribute to gallstone formation, typically due to cholesterol supersaturation and changes in the bile acid pool [2, 3]. Recent studies have increasingly suggested that gut microbiota dysbiosis plays a prominent role in the emergence of these conditions [4]. The gut microbiota helps regulate bile acid and cholesterol metabolism by producing enzymes such as bile acid hydrolase, bile acid hydroxylase, and hydroxy‐methylglutaryl‐CoA (HMG‐CoA) [5, 6, 7, 8]. Only recently, a few researchers have used single bacteria transplantation techniques to analyze the gut microbiota's association with gallstone diseases. These studies have shown that *Desulfovibrionales* members are abundant in the gut of gallstone patients [9]. Additionally, the metabolic product of *Desulfovibrionales*, H_2_S, was observed to increase, inducing hepatic farnesoid X receptor (FXR) and inhibiting CYP7A1 expression. Another study demonstrated that *Lactobacillus* (*L. reuteri* and *L. plantarum*) might relieve CGS through the FXR signaling pathways [10]. To date, cholecystectomy remains the most effective treatment for chronic carriers with gallbladder lithiasis. Ursodeoxycholic acid has been utilized in specific cases to dissolve CGS; however, its use has declined because of the high rates of CGS recurrence [11, 12]. Currently, medical options for preventing and treating CGS are not recommended, and surgical approaches are associated with a significant risk of complications [13, 14]. Therefore, many researchers are focused on finding effective therapies to prevent and treat CGS.

Short‐chain fatty acids (SCFAs), including acetate, butyrate, and propionate, are primary products of dietary fiber fermentation by bacteria. SCFA‐producing bacteria play a role in disease prevention by modulating intestinal inflammation, host immunity, energy metabolism, and insulin sensitivity [15, 16]. As a result, gut microbiota and its metabolites, including SCFAs, hold potential as novel therapeutic targets for gallstones. Fructooligosaccharides (FOS), a form of dietary fiber, influence the composition and function of beneficial gut bacteria, leading to positive physiological effects on the host. FOS has been reported to prevent colitis inflammation by increasing the concentration of 3‐hydroxyoctadecaenoic acid [17]. In addition, FOS supplementation helps regulate gut immune balance and improves glucose and lipid metabolism by promoting the growth of SCFA‐producing microbes in the gut [18, 19, 20]. However, few studies have explored the effectiveness of FOS supplementation in gallstones.

At present, the mechanism of gut microbiota in gallstone pathogenesis is still largely unknown, and the exploration of nonsurgical treatment of gallstones has not achieved significant breakthroughs. The phenotypes and diagnostic features of gut and bile microbiota, along with their metabolites, remain underexplored in the gallstone population, particularly through large‐scale multi‐omics studies. This research identifies the gut microbiome, metabolites, and KEGG orthology (KO) gene profiles associated with CGS and sex‐ and age‐matched healthy controls (HCs). Potential biomarkers were selected based on the metagenomic and metabolomic analysis of stool samples from CGS patients and healthy volunteers matched for both sex and age. Furthermore, the potential intervention effects of specific bacteria strains, SCFAs, and FOS in gallstones‐model mice were explored.

## RESULTS

### Information of the included participants

**Figure 1:**
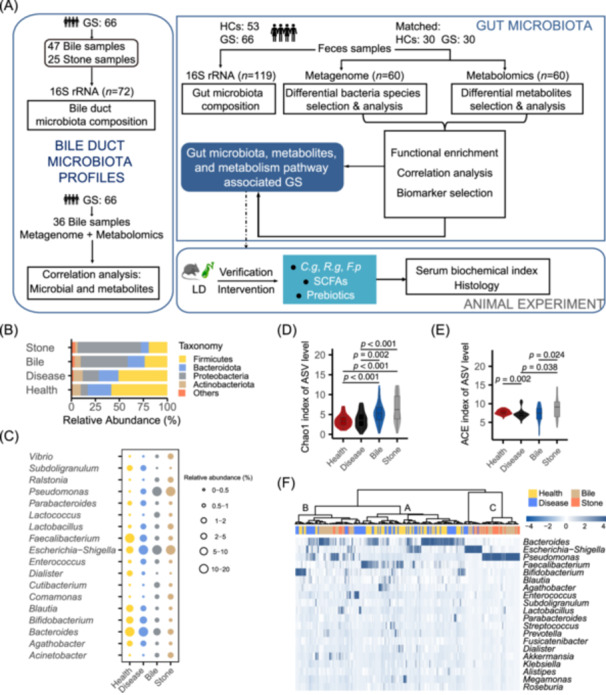
Gut and biliary tract microbiome diversity and structure analysis between CGS and HCs groups. (A) Study design and flow diagram. A total of 191 samples of three types were collected, including 119 fecal samples, 47 bile samples, and 25 stone samples, after rigorous inclusion and exclusion criteria. Bile and fecal samples were detected using UHPLC‐MS/MS to characterize metabolites. Relative abundance of the bacterial community in both groups at the level of phylum (B) and genus (C). Alpha diversity indices of ASVs among groups, Chao1 (D) and ACE (E) index. (F) Heatmap of the relative abundances of top 20 genera for each sample in both groups. ASVs, amplicon sequence variants; CGS, cholesterol gallstone patients; HCs, healthy controls; LD, lithogenic diet; SCFAs, short‐chain fatty acids; UHPLC‐MS/MS, Ultrahigh performance liquid chromatography‐mass spectrometry/mass spectrometry.

A total of 66 subjects with a clinical diagnosis of CGS (average age 56.29 ± 14.18; male: female ratio of 30:36) and 53 HCs (median age 39 [25th–75th percentile range: 29−59]; male: female ratio of 22:31) were recruited from April of 2020 to June of 2021. Meanwhile, age‐ and sex‐matched CGS (*n* = 30) and HCs (*n* = 30) were also selected. The detailed study pipeline is shown in Figure 1A. In the CGS group, compared with HCs, levels of gamma‐glutamyl transpeptidase, high‐density lipoprotein cholesterol (HDL‐C), lymphocytes, monocytes, blood platelets, red blood cells (RBCs), and white blood cells were decreased, while total bilirubin (TB) was increased (all *p* < 0.05). Clinical variables were balanced between the matched groups except for glucose, neutrophils count, and HDL‐C (Table 1).

**Table 1:** Summary of clinical features and laboratory results of participants.

Characteristics	Overall (*n* = 119)	Overall (*n* = 119)			Age‐ and sex‐matched (*n* = 60)		
		Disease (*n* = 66)	Health (*n* = 53)	*p*	Disease (*n* = 30)	Health (*n* = 30)	*p*
Age (years)	52.00 (36.00, 63.00)	56.29 ± 14.18	39.00 (29.00, 59.00)	<0.001	48.00 (37.00, 68.75)	47.00 (36.50, 66.25)	0.796
Gender							
Male	52	30	22	0.806	16	15	1.000
Female	67	36	31		14	15	
BMI (kg/m^2^)	24.88 (22.13, 26.84)	24.93 ± 3.83	25.00 (22.23, 26.35)	0.959	24.81 ± 4.62	24.45 ± 4.12	0.752
GLU (mmol/L)	5.19 (4.90, 5.70)	5.15 (4.80, 5.88)	5.19 (4.90, 5.50)	0.688	5.00 (4.80, 5.27)	5.20 (5.00, 5.68)	0.037
ALT (U/L)	17.00 (11.50, 27.00)	17.50 (12.00, 30.75)	16.00 (11.00, 21.00)	0.158	16.50 (12.00, 30.25)	16.50 (11.00, 20.75)	0.510
AST (U/L)	18.00 (15.00, 22.00)	18.00 (15.00, 22.00)	19.00 (15.00, 22.00)	0.736	16.50 (15.00, 21.50)	19.50 (16.00, 24.25)	0.248
GGT (U/L)	18.00 (11.00, 38.50)	23.00 (13.25, 58.75)	13.00 (10.00, 36.00)	0.010	16.50 (11.50, 31.50)	15.50 (10.00, 40.00)	0.554
HDL‐C (mmol/L)	1.14 (0.93, 1.46)	1.03 (0.92, 1.22)	1.35 ± 0.32	<0.001	1.04 (0.93, 1.21)	1.31 ± 0.33	0.026
LDL‐C (mmol/L)	2.66 (2.10, 3.59)	2.78 (2.12, 3.43)	2.83 ± 0.93	0.808	2.55 ± 0.83	2.88 ± 1.05	0.180
BASO (10^9^/L)	0.03 (0.02, 0.04)	0.03 (0.02, 0.04)	0.03 (0.02, 0.04)	0.162	0.03 (0.02, 0.05)	0.03 (0.02, 0.05)	0.868
EOS (10^9^/L)	0.12 (0.07, 0.20)	0.12 (0.07, 0.22)	0.11 (0.07, 0.16)	0.246	0.14 (0.07, 0.22)	0.11 (0.07, 0.14)	0.304
LYMPH (10^9^/L)	1.94 (1.52, 2.40)	1.74 (1.41, 2.34)	2.16 ± 0.64	0.021	2.01 ± 0.73	2.02 ± 0.53	0.939
MONO (10^9^/L)	0.38 (0.32, 0.47)	0.36 (0.30, 0.45)	0.43 (0.34, 0.50)	0.031	0.40 (0.35, 0.50)	0.44 (0.40, 0.51)	0.274
NEUT (10^9^/L)	3.32 (2.63, 4.20)	3.11 (2.51, 4.23)	3.61 (2.86, 4.19)	0.085	3.14 (2.65, 3.72)	3.85 ± 1.19	0.046
PLT (10^9^/L)	236.00 (190.00, 279.00)	217.50 (169.50, 258.00)	259.81 ± 64.56	0.001	225.00 (198.75, 263.00)	260.50 ± 69.50	0.095
RBC (10^12^/L)	4.47 ± 0.47	4.35 ± 0.42	4.63 ± 0.49	0.001	4.39 ± 0.39	4.60 ± 0.47	0.061
WBC (10^9^/L)	6.05 (4.88, 7.24)	5.78 (4.62, 7.12)	6.54 ± 1.45	0.031	5.93 (4.74, 6.96)	6.40 (5.54, 7.29)	0.167
TB (μmol/L)	11.40 (8.85, 15.10)	12.25 (9.43, 17.45)	11.00 (8.80, 13.00)	0.029	12.25 (9.03, 15.08)	10.41 ± 4.90	0.178
TC (mmol/L)	4.37 ± 0.86	4.26 ± 0.84	4.35 (3.77, 5.04)	0.203	4.02 ± 0.72	4.26 (3.74, 4.98)	0.119
TG (mmol/L)	1.24 (0.82, 1.81)	1.27 (0.95, 1.88)	0.97 (0.75, 1.71)	0.054	1.28 ± 0.57	1.00 (0.76, 1.50)	0.408
Urea (mmol/L)	4.56 (3.88, 5.54)	4.44 (3.80, 5.18)	4.57 (4.05, 5.66)	0.264	4.65 (3.80, 4.91)	5.20 ± 1.19	0.057
URIC (μmol/L)	321.00 (253.00, 397.00)	327.00 (253.75, 417.50)	319.92 ± 98.41	0.506	321.50 (261.00, 430.75)	330.97 ± 108.78	0.579

Abbreviations: ALT, alanine aminotransferase; AST, aspartate aminotransferase; BASO, basophil count; BMI, body mass index; EOS, eosinophil count; GLU, glucose; GGT, gamma‐glutamyl transpeptidase; HDL‐C, high‐density lipoprotein cholesterol; LDL‐C, low‐density lipoprotein cholesterol; LYMPH, lymphocyte count; MONO, monocyte count; NEUT, neutrophil count; PLT, platelets; RBC, red blood cell; TB, total bilirubin; TC, total cholesterol; TG, triglycerides; URIC, uric acid; WBC, white blood cell.

### Biliary tract and gut microbial community

From all the bile, stone, and fecal samples, we identified a total of 3064 amplicon sequence variants (ASVs), distributed as follows: 1016 ASVs in bile, 1955 ASVs in gallstones, 747 ASVs in patient feces, and 683 ASVs in normal feces (Figure S1A). A Venn diagram showed that 172 ASVs were common to all groups, while 1974 ASVs were unique to the gallbladder. Further metagenomic analysis revealed 207 species in bile, 5905 species in patient feces, and 6535 species in normal feces (Figure S1B). At the phylum level, Bacteroidota and Firmicutes were dominant in gut, and Proteobacteria accounted for 50%–60% of the gallbladder microbiota (Figure 1B). Figure 1C displays the relative abundance of genera across the various groups. The top 20 species are shown in Figure S1C. *Escherichia coli*, *Salmonella enterica*, *Streptococcus pneumoniae*, *Klebsiella aerogenes*, and *Chlamydia trachomatis* were dominant in bile. *F. prausnitzii*, *E. coli*, and *Bacteroides vulgatus* were dominant in gut. The Chao1 index was significantly lower in the gut than that in the gallbladder (*p* < 0.05) (Figure 1D). The ACE index was also significantly decreased in CGS fecal samples compared to HCs fecal samples. The ACE index was also significantly decreased in the bile compared to stones (*p* < 0.05) (Figure 1E and Table S1). Our results divided 191 samples into three distinct clusters (A–C) at the genus level, characterized by gut microbiota, the crossover between gut and biliary tract microbiota, and the biliary tract microbiota (Figure 1F).

### 16S rRNA sequencing identified notable differences between CGS group and HCs group

To further investigate the differences in bacterial communities between the CGS group and HCs, 16S rRNA sequencing was applied to fecal samples from 30 CGS patients and 30 age‐ and sex‐matched HCs. The ACE index was significantly lower in CGS fecal samples compared to HCs (*p* < 0.05) (Figure S2A and Table S2). Principle coordinates analysis (PCoA) and ANOSIM test revealed significant differences in microbiome composition and abundance between the two groups (Bray–Curtis distance, *p* = 0.001) (Figure S2B and Table S3). At the genus level, 22 genera showed significant differences in abundance between the two groups, with 12 genera being less abundant in the CGS group (*p* < 0.05) (Figure S2C and Table S4). LEfSe analysis identified 49 discriminative features (LDA > 2, *p* < 0.05) at various taxonomic levels (Figure S2D–E). The linear discriminant analysis (LDA) plot revealed a distinct shift in the microbiota, with CGS individuals showing higher levels of Patescibacteria. Discriminative families in CGS were Pseudomonadaceae, Morganellaceae, Saccharimonadaceae, Vibrionaceae, and Staphylococcaceae. *Pseudomonas*, *Morganella*, *Ruminobacter*, *Faecalibaculum*, *Turicibacter*, *Haemophilus*, and *Ruminococcus* were the key genera that can distinguish CGS from HCs. These genera may serve as potential biomarkers to distinguish gallstone patients from healthy individuals. Among these significantly different genera, 10 genera were further associated with different metabolites (Figure S2F and Table S5). For example, the relative abundance of *Marvinbryantia* was inversely associated with l‐Ascorbic acid 2‐sulfate.

### Metagenomic sequencing revealed potential biomarkers associated with CGS

**Figure 2:**
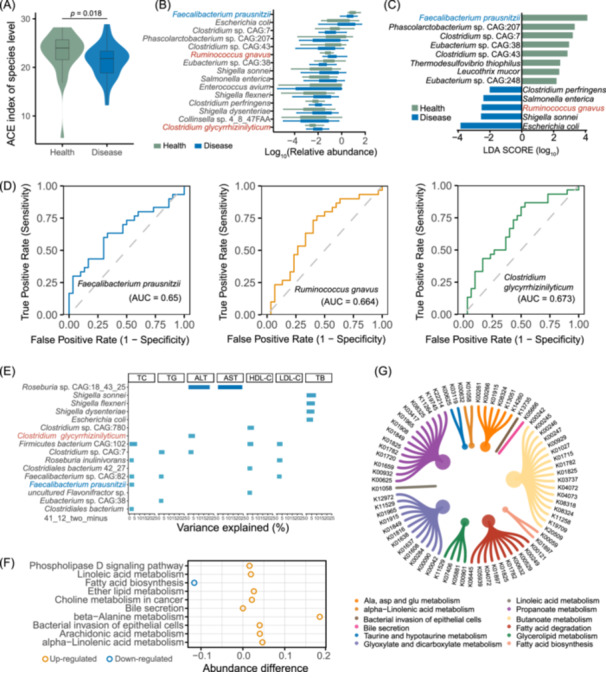
The difference of gut microbiota in CGS and HCs according to the metagenomic data. (A) Alpha diversity ACE index of species between groups. (B) Differential abundance of gut microbiota in CGS and HCs. Green and blue represented the HCs (*n* = 30) and gallstone patients (*n* = 30), respectively. The abundance in each group was plotted as log10 scale on the *y*‐axis. The two‐tailed Wilcoxon rank‐sum test was used to calculate *p*‐values. (C) LDA scores for the bacterial taxa differentially abundant between CGS and HCs (LDA > 3.5). Blue bars indicate taxa were enrichment in CGS, and green bars indicate taxa were enrichment in HCs. (D) Receiver operating characteristic analysis to discriminate CGS from HCs based on strains to be verified (*C. glycyrrhizinilyticum*, *R. gnavus*, and *F. prausnitzii*). (E) The variance of biochemical indices explained by different bacterial species. The height of each bar represented the explained variance calculated using univariate linear regression. The bar represented significant differences (*p* < 0.05). (F) The significant difference relative abundance of each predicted functional category given in the KEGG pathways (level 3). (G) Plot of all alterations KO gene related representative metabolism pathway. Size of circles represents the number of KO identified with an alteration. CGS, cholesterol gallstone patients; HCs, healthy controls.

Metagenomic sequencing was employed on fecal samples from 30 CGS patients and 30 age‐ and sex‐matched HCs to delve deeper into the variance in bacterial species and to identify different KO genes and functions. The ACE index exhibited a significant decrease in CGS fecal samples compared to those of HCs (*p* < 0.05) (Figure 2A). Notably, among the CGS samples, 33 bacterial species, such as *Clostridium perfringens*, *R. gnavus*, and *C. glycyrrhizinilyticum*, showed increased abundance, while 23 bacterial species, including *F. prausnitzii*, *Thermodesulfovibrio thiophilus*, and *Lactobacillus rogosae*, exhibited decreased abundance compared to HCs (Table S6), with representative species shown in Figure 2B.

Additionally, LEfSe was used to generate a cladogram, revealing 31 discriminatory features associated with CGS. 13 discriminative features (LDA > 2, *p* < 0.05) were observed at the species level, including several opportunistic pathogens such as *Escherichia coli*, *Shigella sonnei*, *R. gnavus*, *Salmonella enterica*, and *C. perfringens*, all significantly overrepresented (LDA scores (1og_10_) ≥ 2) in CGS feces, while *F. prausnitzii* dominated in HCs (LDA scores (1og_10_) > 4) (Figure 2C). In the diagnosis of CGS versus HCs, *F. prausnitzii* exhibited a sensitivity of 0.633 and specificity of 0.667, *R. gnavus* had a sensitivity of 0.767 and specificity of 0.567, while *C. glycyrrhizinilyticum* showed a sensitivity of 0.833 and a specificity of 0.500 (Figure 2D). Additionally, several different bacterial species explained a significant proportion of variation in liver function‐related biochemistry traits (e.g., *F. prausnitzii* explained 4.5% of the variance in TC, *C. glycyrrhizinilyticum* explained 6.3% of the variance in ALT) (Figure 2E and Table S7).

Using metagenome sequencing data, we conducted further annotations of functional pathways. The fecal microbiota analysis revealed 36 predictive categories within the KEGG level three functional modules (Table S8). Notably, in the CGS group, activities related to the lipid metabolic pathway, including linoleic acids, arachidonic acids, alpha‐linolenic acids, and ether lipids were observed to be higher (Figure 2F). A total of 1264 KO genes showed significant differences compared to HCs (*p *< 0.05). These significant differences in KO genes were observed in selected representative pathways (Figure 2G), with a majority of differential KO genes being enriched in butanoate and propanoate metabolism, fatty acid degradation, and glyoxylate and dicarboxylate metabolism.

### Metabolic profiling of CGS and HCs

**Figure 3:**
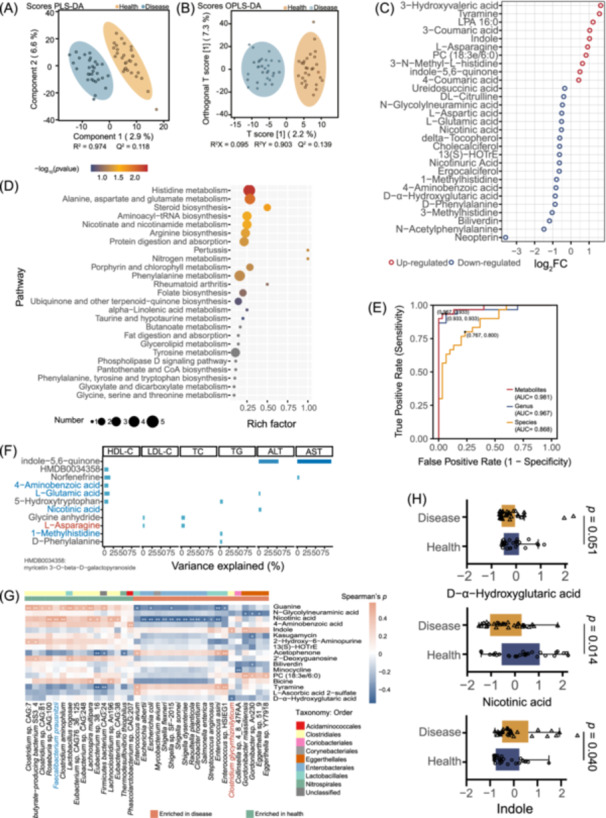
Aberrant metabolic patterns in CGS and HCs. (A, B) The clustering analyses of partial least‐squares discriminant analysis (PLS‐DA) and orthogonal partial least‐squares discriminant analysis (OPLS‐DA). (C) The discriminatory metabolites identified between the CGS and HCs. The *x*‐axis represents the log_2_FC of discriminatory metabolites. Blue and red circles denote CGS decreased and CGS enriched metabolites, respectively. (D) Summary of pathway analysis for 121 differential metabolites in MetaboAnalyst3.0, and significantly enriched pathways are displayed by a bubble plot (*p* < 0.05, Fisher's exact test). (E) Receiver operating characteristic analysis to discriminate CGS from HCs based on species (orange), genus (blue), or metabolites (red) individually. (F) The variance of biochemical indices explained by different metabolites. The height of each bar represented the explained variance calculated using univariate linear regression. The bar represented significant associations (*p* < 0.05). Potential biomarkers are highlighted. Red font: Increased. Blue font: Decreased. (G) Species and metabolites included are those identified as significantly different between CGS and HCs. Enrichment in either group indicated by colored bars to the top of the plot. Significant correlations denoted by white stars (^+^ *p* < 0.1; ^++^ *p* < 0.05, Student's *t*‐test (two‐sided), Benjamini–Hochberg adjustment for multiple comparisons. Exact *p* values are provided in Table S13). Higher taxonomy of species (order) was indicated by colored bars. (H) Levels of D‐α‐Hydroxyglutaric acid, nicotinic acid, and indole. CGS, cholesterol gallstone patients; FC, fold change; HCs, healthy controls.

PLS‐DA and OPLS‐DA analyses showed a distinct metabolic profile between CGS and HCs (Figure 3A,B). Specifically, 76 metabolites, such as indole, PC (18:3e/6:0), and LPA 16:0, were found to be elevated, while 45 metabolites, including nicotinic acid, 4‐Hydroxyisoleucine, and 13(S)‐HOTrE, were reduced in CGS (Table S9). Representative differential metabolites are shown in Figure 3C, which were primarily classified into amino acids, carbohydrates, hydroxycinnamic acids, pyridine carboxylic acids, lipids, pterins and derivatives, and benzenoids. Furthermore, the top 25 enriched metabolic pathways, characterized by significantly different metabolites between CGS and HCs, were identified (Figure 3D and Table S10), including histidine, taurine and hypotaurine, butanoate, and alpha‐linolenic acid metabolism.

We further inspected the individual diagnostic performance of both microbiota and metabolites in distinguishing between CGS and HCs. The area under curve (AUC) values for different bacteria and metabolites ranged from 0.6 to 0.8 in the CGS versus HCs diagnosis. Next, 12 distinctive genera (AUC > 0.7), 8 species (AUC > 0.68), and 13 metabolites (AUC > 0.68) were selected as optimal marker sets (Table S11). Then, combined diagnostic performances for CGS were analyzed. When considering bacterial genus, species, or metabolites alone, AUC values of 96.7%, 86.8%, and 98.1%, respectively, were achieved in discriminating CGS patients from HCs (Figure 3E). To further pinpoint potential biomarkers, we discovered that several metabolites explained a significant proportion of variation in TC, TG, HDL‐C, LDL‐C, ALT, and AST levels. For example, 4‐Aminobenzoic acid and l‐Glutamic acid accounted for 11.0% and 14.2% of the variance in HDL‐C, respectively (Figure 3F and Table S12).

### Correlation analysis between gut microbiota and metabolites

Spearman's correlation analysis was conducted to explore potential associations between the 124 species and 33 metabolites, aiming to uncover links between altered metabolites and gut microbiota (Table S13). The correlation of potential biomarkers in metabolites and various species is shown in Figure 3G. Most of the significantly correlated bacteria belong to the genera *Bifidobacterium*, *Clostridium*, *Shigella*, and *Enterococcus*, which encode bile salt hydrolase (BSH). Several species were significantly correlated with nicotinic acid, PC (18:3e/6:0), and indole, which could originate from the diet or be produced endogenously. Nicotinic acid exhibited negative associations with certain species enriched in CGS, while several species enriched in healthy samples (e.g., *F. prausnitzii*) displayed positive associations, suggesting nicotinic acid as a possible biomarker related to the CGS versus HCs differentiation. The abundance of species such as *Desulfitobacterium chlororespirans*, *Clostridium* sp. CAG:451, *Actinobacteria bacterium* 66_15, positively correlated with the levels of PC (18:3e/6:0). In addition, the abundance of *C. glycyrrhizinilyticum* was positively correlated with the level of indole and was negatively associated with the level of D‐α‐Hydroxyglutaric acid (Figure 3G). The comparison of indole, D‐α‐Hydroxyglutaric acid, and nicotinic acid is shown in Figure 3H.

### Metabolic profiling of bile

A total of 921 compounds in the bile samples were successfully quantified and mapped to the Human Metabolome Database (HMDB). These included 57 lipids and lipid‐like molecules, 54 organic acids and derivatives, and 23 organoheterocyclic compounds (Figure S3A). The top 25 enriched KEGG pathways included steroid hormone biosynthesis and purine metabolism (Figure S3B). To further compare distinct bacterial species between bile and fecal samples from the same gallstone patients, we identified 17 unique species (Figure S3C). Next, we analyzed the correlations of these unique species and metabolites. The abundance of *Neisseria subflava* was negatively correlated with the levels of cholic acid and 7‐Ketolithocholic acid. Similarly, the level of deoxycholic acid showed negative correlations with the abundance of *Staphylococcus saccharolyticus*, *Pseudomonas* sp. 2822‐17, *N. subflava*, *Neisseria polysaccharea*, *Conexibacter woesei*, and *Achromatium* sp. WMS1 (Figure S3D and Table S14).

### Integrative multi‐omics signatures of CGS and HCs

**Figure 4:**
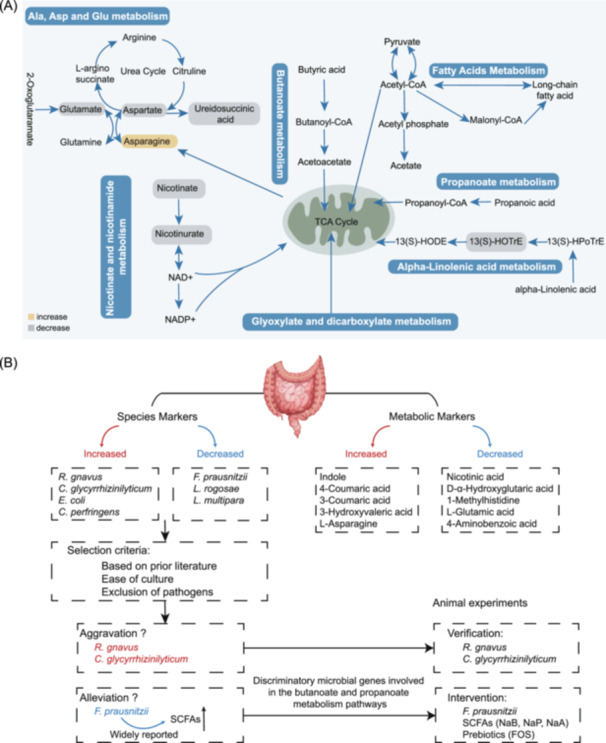
Overview of pathway modules and animal experimental design. (A) CGS‐associated changes in KEGG pathway modules and related different metabolites. KEGG pathway maps including “alpha‐Linolenic acid metabolism,” “Nicotinate and nicotinamide metabolism,” “Alanine, aspartate, and glutamate metabolism,” “Butanoate metabolism,” “Propanoate metabolism,” “Fatty acids metabolism,” and “Glyoxylate and dicarboxylate metabolism.” Each metabolite is marked in yellow for elevation or in gray for depletion. (B) Schematic diagram for animal experiments.

As shown in Figure 4A, we observed various metabolites such as 13(S)‐HOTrE, nicotinate, nicotinurate, l‐glutamate, N‐carbamoyl‐l‐aspartate, l‐asparagine, l‐aspartate, and 1‐methylhistidine, which are involved in alpha‐linolenic acid metabolism, alanine, aspartate, and glutamate metabolism, nicotinate and nicotinamide metabolism, and histidine metabolism. Pathway representations highlighted related differential genes (Table S15). Among the most differentially abundant KO genes, pathways such as butanoate and propanoate metabolism, fatty acid biosynthesis and degradation pathways, taurine and hypotaurine metabolism, and glyoxylate and dicarboxylate metabolism were associated with CGS.

Within the alpha‐linolenic acid metabolism, the gene phospholipase A1 (*pldA*), which hydrolyzes phospholipids to produce 2‐acyl‐lysophospholipids and fatty acids, was notably elevated in CGS (Figure S4). Genes involved in alpha‐linolenic acid metabolism, nicotinate and nicotinamide metabolism, and histidine biosynthesis were significantly elevated in CGS (*p* < 0.05). Phosphate acetyltransferase (*pta*), which participates in acetate assimilation/dissimilation reactions, was significantly reduced in CGS, implicating its involvement in propanoate metabolism and taurine and hypotaurine metabolism (*p* < 0.05).

### *C. glycyrrhizinilyticum* by oral gavaged aggravated gallstone formation

**Figure 5:**
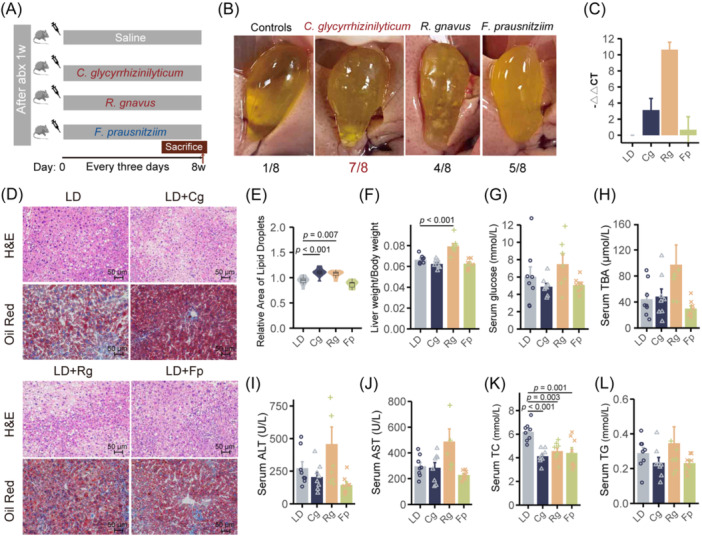
Oral administration of selected species influenced LD‐induced cholesterol gallstones. (A) Schematic diagram for species gavage. (B) Gross appearance of the gallbladders and gallstones of mice in different groups. (C) Abundance of *C. glycyrrhizinilyticum*, *R. gnavus*, and *F. prausnitzii* quantified by qPCR with specific primers. (D) Representative images of H&E‐stained and oil red‐stained liver sections (×200) in different groups. (E) The relative lipid content of each group. Each box centers on the median, with lower and upper bounds representing the first and third quartile (25th and 75th percentile), respectively (*n* = 8). (F) The ratio of liver weight to body weight in different groups. (G, H) Level of serum glucose and serum TBA. (I−L) Levels of serum ALT, AST, TC, and TG. ALT, alanine aminotransferase; AST, aspartate aminotransferase; Cg, *Clostridium glycyrrhizinilyticum*; Fp, *Faecalibacterium prausnitzii*; Rg, *Ruminococcus gnavus*; TC, total cholesterol; TG, triglycerides; TBA, total bile acid. *p*‐values were calculated using one‐way ANOVA with Dunnett's post hoc test.

We selected three bacterial strains for validation, and the screening process is shown in Figure 4B. Subsequently, we subjected antibiotics‐depleted mice to an 8‐week intervention with these bacteria to assess their effects on gallstone formation (Figure 5A). Our findings revealed that the incidence of gallstones was low (12.5%) in the control group, the presence of *C. glycyrrhizinilyticum* significantly increased gallstone formation (87.5%, *p* = 0.010), with visible leaflet crystals or cholesterol particles observed in gallbladders (Figure 5B). Similarly, *R. gnavus* intervention also led to a notable increase in gallstone formation (50%, 4/8). However, gallstone formed in 62.5% (5/8) of the mice receiving *F. prausnitzii* compared to the control group. Real‐time PCR analysis confirmed a significant increase in the abundance of *C. glycyrrhizinilyticum* and *R. gnavus* in the intervention groups compared to the control group (Figure 5C and Table S16).

Further evaluation of histological and biochemical changes revealed marked liver histological damage in the *C. glycyrrhizinilyticum* and *R. gnavus* groups compared to controls (Figure 5D). Oil red O staining confirmed increased lipid droplets in liver parenchyma, particularly in mice from *C. glycyrrhizinilyticum* and *R. gnavus* groups (Figure 5E). Additionally, the liver weight‐to‐body weight ratio was significantly elevated in the *R. gnavus* group (*p* < 0.05) (Figure 5F). Serum glucose and total bile acid (TBA) levels were increased in the *R. gnavus* group compared to controls, though not significantly (Figure 5G,H). Serum alanine aminotransferase (ALT), aspartate aminotransferase (AST), and triglycerides (TG) did not show significant difference among groups. However, serum total cholesterol (TC) levels were significantly decreased in all intervention groups compared to controls (*p* < 0.05) (Figure 5I–L and Table S17).

### FOS supplementation reduced LD‐induced cholesterol gallstones

**Figure 6:**
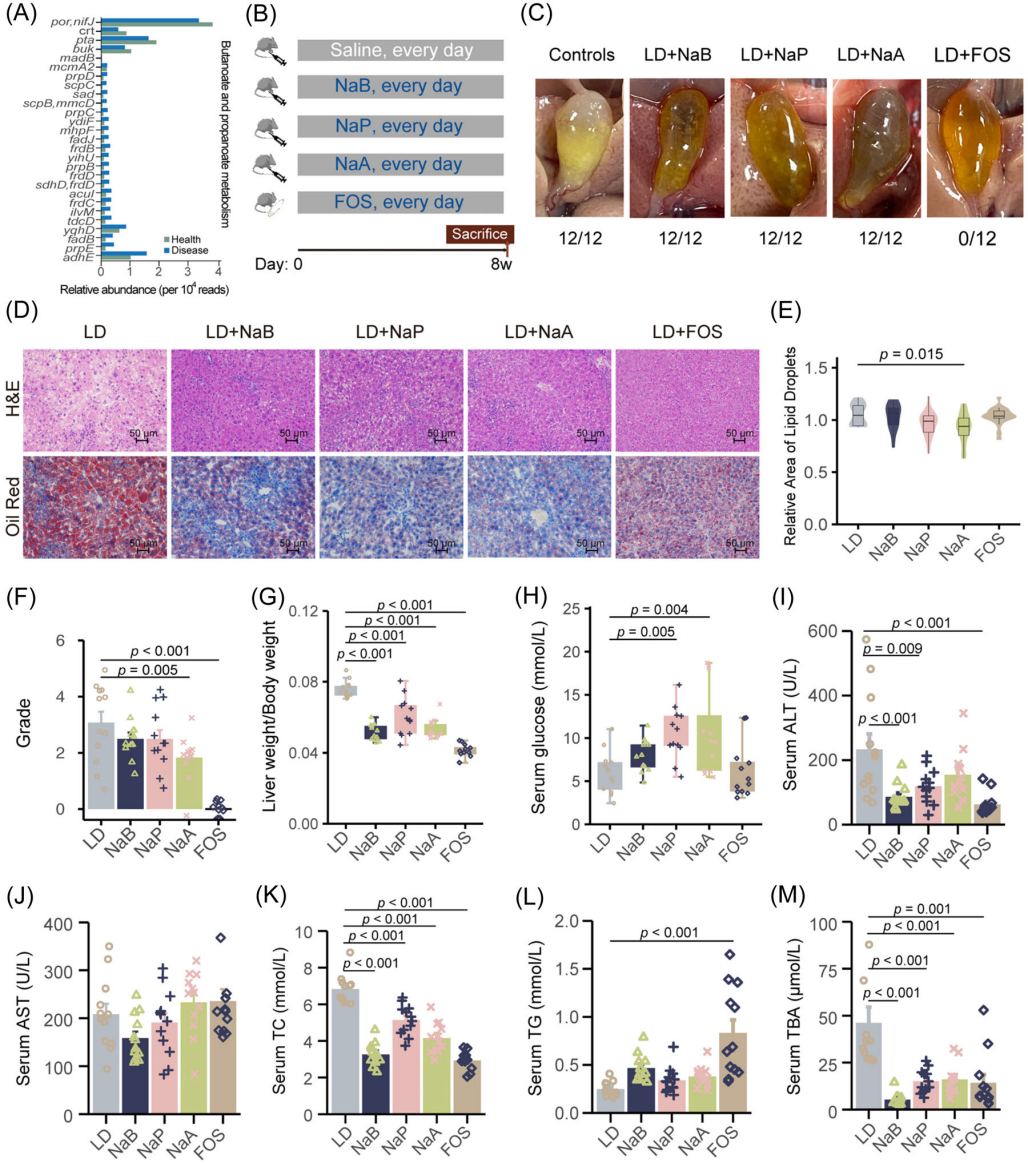
FOS and SCFAs reduced LD‐induced cholesterol gallstones. Twelve mice were randomly assigned to each group and fed a lithogenic diet (LD) with saline, NaB, NaP, NaA, and FOS for 8 weeks (*n* = 12). (A) Relative abundance of the discriminatory microbial genes involved in the butanoate and propanoate metabolism pathways by Wilcox test. (B) Schematic diagram for intervention. (C) Gross appearance of the gallbladders and gallstones of mice in different groups. (D) Representative images of H&E‐stained and oil red‐stained liver sections (×200) in different groups. (E) The relative lipid content of each group. (F) The grade of experimental cholesterol gallstones (CGSs) in the mice was based on the observed gallstone. (G) The ratio of liver weight to body weight in different groups. (H) Level of serum glucose. Each box centers on the median, with lower and upper bounds representing the first and third quartiles (25th and 75th percentile), respectively (*n* = 12). (I−M) Levels of serum ALT, AST, TC, TG, and TBA. ALT, alanine aminotransferase; AST, aspartate aminotransferase; FOS, fructooligosaccharide; NaB, sodium butyrate; NaP, sodium propionate; NaA, sodium acetate; SCFAs, short‐chain fatty acids; TC, total cholesterol; TG, triglycerides; TBA, total bile acid. *p*‐values were calculated using one‐way ANOVA with Dunnett's post hoc test.

Based on the enriched pathways of butanoate and propanoate metabolism, we focused on regulating SCFAs to explore an effective treatment for CGS (Figure 2G). The relative abundance of discriminatory microbial genes involved in the butanoate and propanoate metabolism pathways is shown in Figure 6A. When mice were fed a lithogenic diet (LD), gallstone formation was observed, with 100% incidence at 8 weeks. However, treatment with individual SCFAs, including sodium butyrate (NaB), sodium propionate (NaP), or NaA, did not eliminate cholesterol particles from the gallbladders, although they reduced leaflet crystals or stratified crystals compared to the LD group. Remarkably, mice treated with FOS showed no visible leaflet crystals or cholesterol particles in their gallbladders (0/12) (Figure 6B,C). Furthermore, all treatments protected against liver histological damage, with reduced liver parenchyma lipid accumulation compared to the control group (Figure 6D). Notably, treatment with NaA significantly reduced the relative liver lipid content (*p* < 0.05) (Figure 6E). Grading standards evaluation revealed that the grade of experimental gallstones in control mice was significantly higher than that in mice treated with NaA and FOS (Figure 6F).

Additionally, the liver‐weight to body‐weight ratio was significantly decreased in all treatment groups compared to the control group, with FOS treatment leading to a substantial decrease (*p* < 0.05). Treatment with NaP and NaA significantly increased serum glucose levels (*p* < 0.05). Serum TC and TBA were significantly reduced by all treatments (*p* < 0.05). Moreover, compared to the control group, serum ALT was significantly reduced by NaB, NaP, and FOS treatment (*p* < 0.05). Serum TG was significantly increased in mice treated with FOS (*p* < 0.05), while serum AST was not affected by any treatment (Figure 6G–M and Table S18).

## DISCUSSION

Noninvasive treatments for gallstones remain a clinical challenge. It has been noted that gut microbiota interacts with the host and influences human health through metabolic changes. Multi‐omics studies have identified potential targets related to gallstone formation from various perspectives. Notably, several key bacterial strains such as *F. prausnitzii*, *C. glycyrrhizinilyticum*, and *R. gnavus* have stood out due to their associations with gut health and metabolic diseases [21, 22]. Furthermore, we confirmed that oral gavage of *C. glycyrrhizinilyticum* could exacerbate gallstone formation. To evaluate effective interventions, we conducted experiments on SCFAs and, for the first time, discovered that the intake of FOS could completely inhibit the formation of gallstones induced by LD in mice. Our findings on the microbiota and metabolite profiles offer promising new therapeutic targets for CGS.

Gallstones can currently be detected using various techniques, including abdominal ultrasound, computed tomography (CT), magnetic resonance cholangiopancreatography (MRCP), and endoscopic retrograde cholangiopancreatography (ERCP). Abdominal ultrasonography is simple, cost‐effective, and noninvasive, making it the preferred method; however, it has low sensitivity for small stones and in obese patients. CT scans are expensive and involve radiation exposure. MRCP is highly sensitive but costly, requires contrast agents, and may not be suitable for all patients. ERCP is invasive and carries a risk of complications. Studies have shown that gut microbial biomarkers can differ between CGS and HCs [23, 24, 25]. However, similar studies still face limitations such as small sample sizes and insufficient control for confounding factors such as age and sex. Moreover, due to the high cost of metagenomic sequencing, most research to date has relied on 16S rRNA sequencing, which has limitations in resolving species‐level distinctions and exploring gene functions. Metabolite biomarkers have predominantly been studied in blood and bile samples, with a lack of large‐scale investigations focusing on fecal metabolites [26]. To address these gaps, we systematically analyzed and compared fecal microbial diversity at the species level and explored correlations with clinical indices to identify gut microbial and fecal metabolite biomarkers.

Consistent with previous studies linking phospholipase genes to GS [27, 28], we observed a significant increase in the abundance of *pldA* in CGS. Phospholipase A activity disrupts bile composition by hydrolyzing essential phospholipids, leading to an imbalance that promotes the precipitation of cholesterol and other gallstone‐forming components. Enrichment analysis revealed that the abundance of alpha‐linolenic acid metabolism genes (*pldA*, *fadA*), nicotinate and nicotinamide metabolism genes (*sthA*, *mazG*), and histidine biosynthesis genes (*hisG*, *hisl*) were upregulated in all CGS. Conversely, fatty acid biosynthesis genes (*FabG*, *ACSL*) and butanoate and propanoate metabolism genes (*madB*, *buk*, *OXCT*, *crt*, *PCCA*, *pta*) were downregulated in all CGS. Phosphate acetyltransferase (*pta*), which participates in acetate assimilation/dissimilation reactions, was significantly reduced in CGS and was also involved in the taurine and hypotaurine metabolism. Variations in the gut microbiota and their enzymatic activity may account for these metabolite differences. In addition, glyoxylate and dicarboxylate metabolisms have previously been linked to atherosclerosis and obesity [29, 30]. The glyoxylate cycle can metabolize fatty acids in a glutamate‐dependent manner. In summary, alterations in the gut microbiota influence the host through their metabolic products, involving pathways such as amino acid, fatty acid, and nicotinic acid metabolism. These alterations impact gallstone formation by modulating bile acid conjugation and solubility, exerting anti‐inflammatory effects, and affecting the synthesis, transport, or metabolism of cholesterol [31, 32].

Considering the dominant differential bacterial species, the results of LEfSe analysis, and their contribution to clinical outcome indicators, and after excluding species that are difficult to obtain and culture, we ultimately selected three species. Among the three bacterial species identified in this study, *R. gnavus*, a well‐studied bacterium, has shown a positive association with various diseases, including inflammatory bowel disease, Crohn's disease, and metabolic disorders [33]. However, our results do not provide enough evidence to demonstrate that *R. gnavus* has a significant pro‐stone effect in mice. *C. glycyrrhizinilyticum*, a glycyrrhizin‐hydrolyzing bacterium derived from human feces, is considered a beneficial strain of *Clostridium* due to its various positive effects on the body. [34]. According to Wang et al., the presence of *C. glycyrrhizinilyticum* could elevate serum lipid levels and enhance the inflammatory response in rats with high‐fat diet‐induced nonalcoholic fatty liver disease (NAFLD) [35]. Moreover, liver lipid accumulation was aggravated after *C. glycyrrhizinilyticum* administration, while the levels of serum TC were significantly decreased compared to the LD group. Thus, oral administration of *C. glycyrrhizinilyticum* may promote histological damage in the liver and influence liver lipid metabolism, consequently promoting CGS formation. In addition, *F. prausnitzii* is a potential probiotic, which regulates intestinal health. Although we found improvements in AST, ALT, TC, and TBA after *F. prausnitzii* administration, it still could not significantly inhibit gallstone formation. *F. prausnitzii* typically acts on the host through the production of butyric acid, and we speculate that the level of butyric acid and the duration of the study might have been insufficient to influence the phenotype of gallstones.

In addition, we found that SCFAs metabolism disorder was associated with CGS, which was consistent with previous studies [36, 37]. We found that the grade of experiment stones was significantly decreased in mice by NaA intervention, although the effects between NaP and NaB interventions were not significantly different. Ye et al. demonstrated that NaB mitigates cholesterol gallstones by modulating bile acid metabolism via the FXR‐FGF15/SHP signaling pathway [36]. Their research showed that administering 12 mg/day of NaB for 8 weeks reduced gallstone occurrence from 100% to 25%. The inconsistencies in our present results might be related to injection method, effective dose in vivo, and feed manufacturer, which need further study. Studies have shown that metabolites produced by the gut microbiota, particularly SCFAs, influence liver function and play a significant role in liver diseases such as NAFLD and liver cirrhosis [38, 39]. More studies have explored the roles of prebiotics in regulating SCFAs in metabolic diseases [19, 40, 41]. A recent study showed that resistant starch decreases intrahepatic triglycerides in patients with NAFLD via gut microbiome alterations [40]. Among all prebiotics, FOS can notably increase the level of *F. prausnitzii*. We also found the effectiveness of FOS in mice with a significantly decreased level of serum TC, ALT, and TBA. Therefore, we hypothesize that FOS supplements may alleviate cholesterol gallstones by increasing SCFAs‐producing bacteria. Hu et al. reported the prepared pectin oligosaccharides (POS) exhibited hypocholesterolemic effects that were modulated by specific bacterial groups together with their metabolites [41]. Besides, Huang et al. recently reported that FOS attenuated NAFLD via regulating lipid metabolism [19]. Based on these studies, we assume that FOS may increase SCFAs levels by regulating the gut microbiota composition and that SCFAs play a beneficial role in preventing gallstones by regulating cholesterol metabolism and inhibiting the accumulation of lipids in the liver. Among the prebiotics currently recognized as safe natural food constituents, there are some concerns with the side effects, such as flatulence [42], jaundice, and cholestasis [43]. Singh et al. reported that feeding soluble fibers for 6 months caused cholestatic liver cancer in mice with an imbalance of intestinal flora [44]. Importantly, no significant side effects of FOS were noted in our study with mice. However, as the experiment lasted only 8 weeks, a longer duration is necessary to further validate these findings.

The innovations in our study include the use of multi‐omics data for discovery and phenotypic verification. These data helped us to expand the lithiogen species spectrum. We also have demonstrated for the first time in mice models that dietary FOS is effective in preventing stone development. Despite these meaningful findings, as a cross‐sectional study, some changes in bacterial composition and metabolites might have been caused by confounding factors (e.g., diet, medication history), which need to be clarified through mechanistic experiments in our future work. Although our research findings did not delve into the mechanisms, the current results serve as a springboard for further investigations, aiming to stimulate more research on microbial‐targeted interventions involving probiotics and prebiotics and identify effective preventive and noninvasive treatment modalities for patients with gallstones.

This study has some limitations. First, we focused primarily on the preventive rather than the therapeutic effects of FOS on CGS, examining its protective role against stones in animals. Further studies are needed to explore both the preventive and therapeutic effects of dietary fiber oligosaccharides in clinical patients. Second, the effect of SCFA interventions did not completely inhibit stones, and whether it is related to the intervention time and dose needs further research. Third, we used male mice in this study, and the potential impact of sex on the results has not been assessed. Fourth, the role of *C. glycyrrhizinilyticum* should be further explored in germ‐free mice. Finally, the molecular mechanism disturbing the cholesterol metabolism by FOS needs to be further elucidated.

## CONCLUSION

*C. glycyrrhizinilyticum* carriage promotes gallstone formation. FOS can be a new, cost‐effective, and relatively simple microbiota‐targeted therapeutic approach for CGS. Our findings could help deepen the understanding of CGS pathogenesis and pave the way for the development of novel microbiome‐based treatments or microbiota‐targeted foods.

## METHODS

### Subjects and design

The study was approved by the Ethics Committees of Beijing Hospital (approval number: 2021BJYYEC‐243‐01). Informed consent forms have been collected from this study's participant, who provided their feces, bile, and stone samples. This study recruited 66 gallstone patients and 53 HCs from the Department of Surgery of Beijing Hospital affiliated from April 1, 2020, to June 1, 2021. A total of 72 samples (47 bile samples and 25 stone samples) were obtained from these 66 patients during cholecystectomy. We collected clinical metadata, including age, sex, BMI, glucose, blood routine, and biochemical indicators. HCs were recruited through advertisements and were also assessed with ultrasonography to ensure that they did not have gallstones. The inclusion criteria of the patients were as follows: (a) having undergone ultrasonography; (b) with a diagnosis of gallstone; and (c) understood the study and signed the informed consent form. The exclusion criteria included: (a) with severe gastrointestinal diseases (inflammatory bowel diseases, cancer, and/or advanced adenoma); (b) using immunosuppressive agents or antibiotics within 30 days or probiotic products within 14 days; (c) with metabolic disorders, such as obesity, diabetes mellitus, hyperlipidemia, chronic bowel disease, chronic diarrhea, and/or constipation; (d) long‐term use of contraceptive drugs; (e) pregnant women; and (f) current and ex‐smokers. Participants were supplied with a fecal collection kit and instructed to collect feces.

### Microbiota analysis

DNA extraction and sequencing methods are detailed in online supplementary materials. Alpha diversity index (such as observed ASVs, Chao1, Shannon, and Simpson) took into account the number and evenness of the bacteria in a sample. PCoA was used to interrogate the robustness of group‐wise clustering, and the Adonis test was used to estimate group‐wise beta diversity. Diversity and richness analyses were done with R 4.1.2 using the vegan package (V.2.6‐4). The 30 gallstone participants and their sex and aged‐matched controls were picked out to perform difference analysis. Bacterial species with a mean relative abundance of ≥0.001% combined with a prevalence of ≥10% was selected for difference analysis.

### Mice experiments

Male C57BL/6J mice were bought from SPF Biotechnology Co. Ltd and were used to induce a mouse model of gallbladder gallstones (20–22 g, SPF grade). Male mice (at the age of 8 weeks) were fed with LD (containing 1.25% cholesterol and 0.5% cholic acid, synthesized by Xietong Pharmaceutical Bio‐engineering Co. Ltd, Jiangsu, China) for 8 weeks before sacrifice. The mice were bred on a 12‐h light/12‐h dark cycle in a controlled temperature (22.5 ± 2.5°C) and humidity (50 ± 5%) environment. All experimental procedures were approved by the Institutional Animal Care and Use Committee (IACUC) of Peking University Health Science Center (PUIRB‐LA2023004, No. BCJG0185).

In bacteria transplantation study, *Ruminococcus gnavus* (Rg, BNCC319000), *C. glycyrrhizinilyticum* (Cg, JCM‐13368), and *Faecalibacterium prausnitzii* (Fp, DSM‐107838) were purchased by BeNa Culture Collection Co. Ltd. (Beijing, China), and cultured under strict anaerobic conditions overnight. C57BL/6J mice were depleted of gut microbes by antibiotics (Abx) cocktails (containing 0.5 g/L vancomycin, 1 g/L neomycin sulfate, 1 g/L metronidazole, 1 g/L ampicillin) in drinking water for 1 week, meanwhile, oral gavaged once per day. Then, the mice were orally gavaged with 200 μL sterile normal saline or live Rg, Cg, or Fp at a dose of 10^9^ colony‐forming units/mL in sterile PBS, respectively, four times a week for 3 weeks. Upon starting of gavage, mice were fed with LD for 8 weeks (*n* = 8 mice/group).

In SCFAs intervention study, sodium acetate (NaA, S818278, Macklin), sodium propionate (NaP, S817368, Macklin), and sodium butyrate (NaB, S817488, Macklin) have been delivered to mice via intraperitoneal (IP) injection. To determine the effect of SCFAs, the mice fed an LD received 200 μL of NaA solutions (300 mg/kg), NaP (300 mg/kg), and NaB (300 mg/kg) for 8 weeks, respectively. The LD group received an equal volume of normal saline to serve as the control (*n* = 12 mice/group).

In the FOS intervention study, the experiment group was given a standard chow diet supplemented with 10% FOS for 1 week and then given LD supplemented with 10% FOS (Quantum Hi‐Tech Biological Co. Ltd.) for 8 weeks. The control group was given the same diet without FOS (*n* = 12 mice/group).

All mice were fasted 8 h before sacrifice. The grading standards developed by Akiyoshi and colleagues based on the cholesterol content were used to evaluate the grade of gallstones [45]. The blood sample was obtained by heart puncture and centrifuged at 900 *g* for 10 min at 4°C for subsequent biochemical analysis. Serum biochemical indicators were measured by a Hitachi fast automatic biochemical analyzer (LABOSPECT 008 AS). Liver and cecal contents were collected and stored at −80°C under analysis.

### Histology

Fresh liver specimens were fixed in 4% neutral paraformaldehyde at room temperature for 24 h. Paraffin‐embedded sections (4 μm) were stained with hematoxylin and eosin (H&E). Oil‐red O staining was used to measure hepatic lipid accumulation. Frozen liver sections (6 μm) were stained with oil red O lipid stain, and the relative lipid content was quantified by ImageJ.

### Statistical analysis

Statistical analyses were performed using R 4.1.2 software. Continuous variables were expressed as mean ± standard deviation (SD) for normally distributed data, and as median with interquartile range (IQR) for non‐normally distributed data. The Shapiro–Wilk test was used to assess normality. Differences between groups were analyzed using an unpaired Student's *t*‐test for normally distributed variables and a Wilcoxon rank‐sum test for non‐normally distributed variables. For multiple group comparisons, one‐way analysis of variance (ANOVA) was used for normally distributed data, followed by Dunnett's post hoc test, while the Kruskal‐Wallis test was applied for non‐normally distributed data, with Dunn's test for pairwise comparisons. Categorical data were analyzed by the chi‐square test or Fisher's exact test. Data process and visualization were performed by R packages including dplyr, readr, stringr, ggplot2, ggsci, aPCoA, pheatmap, ggraph, corrplot, psych, DESeq. 2, vegan, and ggsignif. Differential abundance of phyla, genera, species, and functional modules between any two groups was tested by Wilcoxon rank‐sum test, *p*‐value was corrected as false discovery rate with the Benjamini–Hochberg method. The variation of liver function‐related biochemistry traits explained by gut bacterial species and metabolites was calculated using univariate linear regression. Spearman's correlation analysis was performed to analyze the correlations between clinical index or metabolites and bacterial species. The thresholds of Spearman correlations were *rs* > 0.7 and *p* ≤ 0.05 and visualized by heatmap. The univariate and multivariable logistic regression model was used to evaluate diagnostic performance, and the area under the receiver operating characteristic curve was calculated. A *p* < 0.05 or *q* < 0.1 was considered as statistically significant.

## AUTHOR CONTRIBUTIONS

**Ye Liu**: Writing—original draft; writing—review and editing; visualization; formal analysis; data curation; software; methodology. **Hexin Li**: Resources; investigation; data curation. **Tianhan Sun**: Methodology; software. **Gaoyuan Sun**: Methodology; investigation; data curation. **Boyue Jiang**: Methodology; software. **Meilan Liu**: Data curation; software. **Qing Wang**: Data curation; investigation. **Tong Li**: Investigation; software. **Jianfu Cao**: Validation; data curation. **Li Zhao**: Validation; methodology; software. **Fei Xiao**: Conceptualization; writing—review and editing; project administration. **Fangqing Zhao**: Conceptualization; project administration; writing—review and editing. **Hongyuan Cui**: Resources; project administration; conceptualization; writing—review and editing.

## CONFLICT OF INTEREST STATEMENT

The authors declare no conflicts of interest.

## ETHICS STATEMENT

The study was approved by the Ethics Committees of Beijing Hospital (approval number: 2021BJYYEC‐243‐01). All experimental procedures were approved by the Institutional Animal Care and Use Committee (IACUC) of Peking University Health Science Center (PUIRB‐LA2023004, No. BCJG0185). All subjects provided informed consent to participate.

## Supporting information


**Figure S1.** Gut and biliary tract microbiome community.
**Figure S2.** The difference of gut microbiota in CGS (*n* = 30) and HCs (*n* = 30) according to the 16S rRNA data.
**Figure S3.** Conjoint analysis of microbiota and metabolites in bile samples.
**Figure S4.** Genes possibly associated with gallstones in previous studies.

**Table S1.** Alpha diversity indices of ASVs among groups based on 16S rRNA sequencing.
**Table S2.** Alpha diversity indices of age‐ and sex‐matched samples' gut microbiota at ASVs levels based on 16S rRNA sequencing.
**Table S3.** Principal coordinate analysis (PCoA) of the gut microbiota based on the bray‐curtis distance metrics for CGS and matched HCs by 16S rRNA sequencing.
**Table S4.** Different bacteria of the gut microbiota at the genus level for CGS and matched HCs by 16S rRNA sequencing (Wald test, FDR‐corrected *q* < 0.05, and fold change > 2).
**Table S5.** Correlations between selected different metabolites and different genera (matched samples).
**Table S6.** Relative abundance of different species between CGS and HCs groups.
**Table S7.** The variance of biochemical indices explained by different bacterial species.
**Table S8.** Relative abundance of different KEEG pathways at level 3.
**Table S9.** 121 different metabolites for CGS and matched HCs.
**Table S10.** Significantly enriched pathways by 121 differential metabolites.
**Table S11.** Receiver operating characteristic (ROC) analysis to discriminate CGS from HCs individually.
**Table S12.** The variance of biochemical indices explained by different metabolites.
**Table S13.** Correlations between selected different metabolites and different species.
**Table S14.** Correlations between bile metabolites and bile unique species.
**Table S15.** CGS‐associated changes in microbial genes summarized in KO genes and KEGG pathway modules.
**Table S16.** qPCR CT values.
**Table S17.** Animal clinical indicators of single bacteria transplantation.
**Table S18**. Animal clinical indicators of FOS and SCFAs intervention groups.

## Data Availability

The raw sequence data reported in this paper have been deposited to the China National Center for Bioinformation/Beijing Institute of Genomics, Chinese Academy of Sciences (GSA‐Human: HRA004238) that are publicly accessible at https://bigd.big.ac.cn/gsa-human/browse/HRA004238. The metabolome data reported in this paper have been deposited in the OMIX (accession no. OMIX003481), China National Center for Bioinformation/Beijing Institute of Genomics, Chinese Academy of Sciences (https://download.cncb.ac.cn/OMIX/OMIX003481/). The data and scripts used are saved in GitHub (https://github.com/rhyer0418/CGS-project). Supplementary materials (methods, figures, tables, graphical abstract, slides, videos, Chinese translated version, and update materials) may be found in the online DOI or iMeta Science http://www.imeta.science/.
