# Supplementary material for: Gut microbiome and metabolome characteristics of patients with cholesterol gallstones suggest the preventive potential of prebiotics
Source: Imeta. 2025 Feb 21;4(1):e70000. doi: 10.1002/imt2.70000 (PMC11865347; doi:10.1002/imt2.70000)
Supplement: Supplementary file 1 — Figure S1. Gut and biliary tract microbiome community. Figure S2. The difference of gut microbiota in CGS (n = 30) and HCs (n = 30) according to the 16S rRNA data. Figure S3. Conjoint analysis of microbiota and metabolites in bile samples. Figure S4. Genes possibly associated with gallstones in previous studies. [file IMT2-4-e70000-s002.docx]

**Supporting information to**

**Gut microbiome and metabolome characteristics of patients with cholesterol gallstones suggest the preventive potential of prebiotics**

Ye Liu^1,2,3^, Hexin Li^2^, Tianhan Sun^4^, Gaoyuan Sun^2^, Boyue Jiang^4^, Meilan Liu^4^, Qing Wang^4^, Tong Li^4^, Jianfu Cao^4^, Li Zhao^5^, Fei Xiao^1,2,3*^, Fangqing Zhao^6,7*^, Hongyuan Cui^4*^

^1^Peking University Fifth School of Clinical Medicine, Beijing Hospital, National Center of Gerontology, Beijing, 100730, China

^2^Clinical Biobank, Beijing Hospital, National Center of Gerontology, Institute of Geriatric Medicine, Chinese Academy of Medical Sciences, Beijing, 100730, China

^3^The Key Laboratory of Geriatrics, Beijing Institute of Geriatrics, Institute of Geriatric Medicine, Chinese Academy of Medical Sciences, Beijing Hospital/National Center of Gerontology of National Health Commission, Beijing, 100730, China

^4^Department of General Surgery, Beijing Hospital, National Center of Gerontology, Institute of Geriatric Medicine, Chinese Academy of Medical Sciences, Beijing, 100730, China

^5^Department of Gastroenterology, Beijing Hospital, National Center of Gerontology, Institute of Geriatric Medicine, Chinese Academy of Medical Sciences, Beijing, 100730, China

^6^Institute of Zoology, Chinese Academy of Sciences, Beijing 100101, China

^7^Key Laboratory of Systems Biology, Hangzhou Institute for Advanced Study, Beijing Institutes of Life Science, Chinese Academy of Sciences, Beijing 100101, China

*Correspondence: [cuihongyuan3921@bjhmoh.cn](mailto:cuihongyuan3921@bjhmoh.cn) (Hongyuan Cui), zhfq@biols.ac.cn (Fangqing Zhao), [xiaofei3965@bjhmoh.cn](mailto:xiaofei3965@bjhmoh.cn) (Fei Xiao)

**Methods**

## DNA extraction

Fecal samples were collected in sterile plastic pots the morning of the surgery or the previous evening and kept at −80°C until DNA extraction. DNA was extracted by using a magnetic soil and stool DNA kit (#DP712, Tiangen, Beijing, China) according to the instructions of the manufacturer. Bile and gallstone samples were collected in sterile tubes after the surgery and kept at −80°C until DNA extraction. DNA from bile and gallstone was extracted using CTAB method. DNA concentration and purity was monitored on 1% agarose gels. DNA was diluted to 1ng/μL using sterile water.

## 16S rRNA gene sequencing

The 16S rRNA gene encompassing the V3−V4 regions were targeted using the 341F (5′- CCTAYGGGRBGCASCAG-3′) and 806R (5′- GGACTACNNGGGTATCTAAT-3′) primers. Amplicons were extracted from 2% agarose gel, purified by the Qiagen Gel Extraction Kit (Qiagen, Germany). Following manufacturer’s recommendations, sequencing libraries were generated with NEBNext^®^ Ultra™ IIDNA Library Prep Kit (NEB, USA). The library quality was assessed on the Qubit@ 2.0 Fluorometer (Thermo Scientific) and Agilent Bioanalyzer 2100 system. At last, the library was sequenced on an Illumina NovaSeq6000 platform (Novogene, China) and 250 bp paired-end reads were generated.

Raw fastq files were merged using FLASH (Version 1.2.11) parameters: -m 10 -f 300 -x 0.1 -p 33 -r 199 -M 173. Paired-end reads was assigned to each sample according to the unique barcodes. Sequences were analyzed using Quantitative Insights into Microbial Ecology 2 software (QIIME2, Version 2020.6) with default settings [1]. The merged reads were processed through the DADA2 to reduce noise, filter chimeric reads, and obtain the amplicon sequence variant (ASV) feature sequences [2]. Taxonomies were assigned using the SILVA (Version 138.1) as the reference database [3]. All 16S rRNA raw data have been submitted to GSA-Human Database (accession number: HRA004238) [4,5].

## Metagenomic sequencing

About 1μg DNA per sample were prepared. The genomic DNA was randomly sheared into fragments of approximately 350 bp, which were used for library construction using NEBNext^®^ Ultra™ DNA Library Prep Kit for Illumina (NEB, USA). The obtained fragments were end repaired, A-tailed and further ligated with Illumina adapter. The fragments with adapters were PCR amplified, size selected, and purified. The library was checked with Qubit and real-time PCR for quantification and bioanalyzer for size distribution detection. The quantified library was pooled and sequenced using 2 × 150 bp paired-end sequencing on an Illumina HiSeq-platform at the Novogene Bioinformatics Technology Co., Ltd. (Tianjin, China). All metagenomic raw data have been submitted to GSA-Human Database (accession number: HRA004238) [4,5].

Quality control and preprocessing of raw FASTQ reads were performed using kneaddata (V0.12.0) and Bowtie2 (v2.5.1) software. These steps included read trimming, adapter removal and separation of both rRNA sequences (SILVA V.128) and human host DNA (Homo_-sapiens. GRCh38). Metagenomics data were assembled using MEGAHIT (v1.2.9), and contigs with the length being or more than 300 bp were selected as the final assembling result [6]. The reads were aligned using DIAMOND (v0.9.9) for taxonomy functional assignment and taxonomic identity. The LCA algorithm of MEGAN software system was used to conduct annotation [7]. The genes were predicated using the MetaGeneMark (v2.10). Redundant genes (95% identity, 90% overlap) were removed by CD-HIT ([v](http://www.bioinformatics.org/cd-hit)4.5.8) [8], resulting in a non-redundant gene catalog. Predicted unigenes were used DIAMOND to assign to the kyoto encyclopedia of genes and genomes (KEGG). The abundances of each functional annotations were the sum of the abundance of annotation of each functional level.

## Metabolite extraction, profiling and analysis

Fecal specimens (100 mg) were individually grounded with liquid nitrogen and the homogenate was resuspended with prechilled 80% methanol by well vortex. The bile samples (100 μL) were placed in the EP tubes and resuspended with prechilled 80% methanol by well vortex. The samples were then incubated on ice for 5 min and centrifuged at 15,000 g, 4°C for 20 min. Some of supernatant was diluted to final concentration containing 53% methanol by liquid chromatography-mass spectrometry (LC-MS) grade water. The samples were subsequently transferred to a fresh Eppendorf tube and then were centrifuged at 15,000 g, 4°C for 20 min. Finally, the supernatant was injected into the UHPLC-MS/MS system analysis. Quality control (QC) samples were prepared by equally pooling 10 samples from the same batches, which were injected at designated intervals.

UHPLC-MS/MS analyses were performed using a Vanquish UHPLC system (Thermo Fisher, Germany) coupled with an Orbitrap Q Exactive^TM^ HF mass spectrometer (Thermo Fisher, Germany) in Novogene Co., Ltd. (Beijing, China). Samples were injected onto a Hypesil Gold column (100 × 2.1 mm, 1.9 μm) using a 17-min linear gradient at a flow rate of 0.2 mL/min. The eluents for the positive polarity mode were eluent A (0.1% FA in Water) and eluent B (Methanol). The mobile phases for negative mode were A (5 mM ammonium acetate, pH 9.0) and B (Methanol). The solvent gradient was set as follows: 2% B, 1.5 min; 2%–100% B, 3 min; 100% B, 10 min; 100%–2% B, 10.1 min; 2% B, 12 min. MS detection was operated in positive/negative polarity mode with spray voltage of 3.5 kV, capillary temperature of 320°C, sheath gas flow rate of 35 psi and aux gas flow rate of 10 L/min, S-lens RF level of 60, and aux gas heater temperature of 350°C.

The raw data files generated by UHPLC-MS/MS were processed using the Compound Discoverer 3.1 (CD3.1, Thermo Fisher) to perform peak alignment, peak picking, and quantitation for each metabolite. The main processing parameters were set as follows: retention time tolerance, 0.2 min; mass tolerance, 5ppm; signal intensity tolerance, 30%; signal/noise ratio, 3; and minimum intensity, 100,000. After that, peak intensities of metabolites were normalized to the total spectral intensity, which was used to predict metabolites based on additive ions, molecular ion peaks and fragment ions. And then peaks were matched with the mzCloud (<https://www.mzcloud.org/>), mzVault and MassList database to obtain the accurate qualitative and relative quantitative results.

Identified metabolites were annotated using the KEGG database (<https://www.genome.jp/kegg/pathway.html>), HMDB database (<https://hmdb.ca/metabolites>) and Lipidmaps database (<http://www.lipidmaps.org/>). Partial least squares discriminant analysis (PLS-DA) and orthogonal PLS-DA (OPLS-DA) were performed using R. The metabolites with VIP > 1 and *p* (*t*-test) < 0.05 and fold change (FC) ≥ 2 or FC ≤ 0.5 were considered to be differential metabolites. The functions of these metabolites and metabolic pathways were studied using the KEGG database. The metabolic pathway enrichment of differential metabolites was performed using MetaboAnalyst ([https://www.metaboanalyst.ca/) and](https://www.metaboanalyst.ca/)%20and) the abundance of metabolites was normalized by Autoscaling.

## Metabolomic and metagenomic data integration

Correlation analysis between metagenomic and metabolomic data was undertaken using genomes and metabolites identified as significantly different between CGS and healthy samples. The co-occurrence was calculated on the basis of the relative abundances by Spearman’s rank correlation coefficient (*p* < 0.05). The network layout was calculated and visualized using a circular layout by the Cytoscape software [9]. Only edges with correlations greater than 0.5 were shown, and unconnected nodes were omitted. Correlation coefficients with a magnitude of 0.5 or above were selected for visualization in Cytoscape (version 3.9.1).

## Real-time quantitative polymerase chain reaction

To evaluate the colonization of single strains after oral administration, we collected cecal contents from the mice. The DNA was extracted using MagPure Stool DNA Kit (D6364-01, Magen, Guangdong, China) according to the manufacturers’ instructions. Quantitative real-time PCR was performed using SYBR Green master mix on qPCR system (Archimed X4, ROCGENE, Xuzhou, China). The PCR program was optimized to ensure no positive amplification for these baseline samples. Positive control with DNA of each target strain and negative control using water instead of genomic DNA were included in all PCR runs. The PCR system (20 μL) consisted of 1 μL genomic DNA (template), 10 μL 2× premix (AGbio, Hunan, China), 1 µL forward primer (0.2 µmol in the final mixture), 1 µL reverse primer (0.2 µmol in the final mixture), and 7 µL ddH_2_O. The following qPCR program yielded the most selective amplification: an initial denaturation step at 95°C for 1 min; 40 cycles of denaturation at 95°C for 15 s, and annealing/extension at 60°C for 30 s; a melt curve analysis between 60°C and 95°C in 0.5°C increments at 2–5 s/step; and polymerase activation and DNA denaturation at 95°C for 5 min. Relative changes in bacteria strains were calculated using the 2^-ΔΔCT^ method. The primer sequences used were as follows: for *F. prausnitzii*, forward primer 5′-GGAGGAAGAAGGTCTTCGG-3′ and reverse primer 5′-AATTCCGCCTACCTCTGCACT-3′ [10]; for *R. gnavus*, forward primer 5′-GGACTGCATTTGGAACTGTCAG-3′ and reverse primer 5′-AACGTCAGTCATCGTCCAGAAAG-3′ [11]; and for *C. glycyrrhizinilyticum*, forward primer 5′-GACCGCTTGAAATGGGTGTC-3′ and reverse primer 5′-GCGGACAAGGTAAAGCTCCT-3′. Primers designed by <https://www.ncbi.nlm.nih.gov/tools/primer-blast/>.

**References**

1. Bolyen, Evan, Jai Ram Rideout, Matthew R. Dillon, Nicholas A. Bokulich, Christian C. Abnet, Gabriel A. Al-Ghalith, Harriet Alexander, et al. 2019. “Reproducible, interactive, scalable and extensible microbiome data science using QIIME 2.” *Nature Biotechnology* 37: 852−857. https://doi.org/10.1038/s41587-019-0209-9

2. Callahan, Benjamin J., Paul J. McMurdie, Michael J. Rosen, Andrew W. Han, Amy Jo A. Johnson, Susan P. Holmes. 2016. “DADA2: High-resolution sample inference from Illumina amplicon data.” *Nature Methods* 13: 581−583. https://doi.org/10.1038/nmeth.3869

3. Quast, Christian, Elmar Pruesse, Pelin Yilmaz, Jan Gerken, Timmy Schweer, Pablo Yarza, Jörg Peplies, Frank Oliver Glöckner. 2013. “The SILVA ribosomal RNA gene database project: improved data processing and web-based tools.” *Nucleic Acids Research* 41: D590−D596. https://doi.org/10.1093/nar/gks1219

4. Chen, Tingting, Xu Chen, Sisi Zhang, Junwei Zhu, Bixia Tang, Anke Wang, Lili Dong, et al. 2021. “The genome sequence archive family: toward explosive data growth and diverse data types.” *Genomics, Proteomics & Bioinformatics* 19: 578−583. https://doi.org/10.1016/j.gpb.2021.08.001

5. Members, Cncb-Ngdc, Partners. 2022. “Database resources of the national genomics data center, china national center for bioinformation in 2022.” *Nucleic Acids Research* 50: D27−D38. https://doi.org/10.1093/nar/gkab951

6. Li, Dinghua, Chi-Man Liu, Ruibang Luo, Kunihiko Sadakane, Tak-Wah Lam. 2015. “MEGAHIT: an ultra-fast single-node solution for large and complex metagenomics assembly via succinct de Bruijn graph.” *Bioinformatics* 31: 1674−1676. https://doi.org/10.1093/bioinformatics/btv033

7. Huson, Daniel H., Suparna Mitra, Hans-Joachim Ruscheweyh, Nico Weber, Stephan C. Schuster. 2011. “Integrative analysis of environmental sequences using MEGAN4.” *Genome Research* 21: 1552−1560. https://doi.org/10.1101/gr.120618.111

8. Li, Weizhong, Adam Godzik. 2006. “Cd-hit: a fast program for clustering and comparing large sets of protein or nucleotide sequences.” *Bioinformatics* 22: 1658−1659. https://doi.org/10.1093/bioinformatics/btl158

9. Shannon, Paul, Andrew Markiel, Owen Ozier, Nitin S. Baliga, Jonathan T. Wang, Daniel Ramage, Nada Amin, Benno Schwikowski, Trey Ideker. 2003. “Cytoscape: a software environment for integrated models of biomolecular interaction networks.” *Genome Research* 13: 2498−2504. https://doi.org/10.1101/gr.1239303

10. Fitzgerald, Cormac Brian, Andrey N. Shkoporov, Thomas D. S. Sutton, Andrei V. Chaplin, Vimalkumar Velayudhan, R. Paul Ross, Colin Hill. 2018. “Comparative analysis of Faecalibacterium prausnitzii genomes shows a high level of genome plasticity and warrants separation into new species-level taxa.” *BMC Genomics* 19: 931. https://doi.org/10.1186/s12864-018-5313-6

11. Png, Chin Wen, Sara K Lindén, Kristen S Gilshenan, Erwin G Zoetendal, Chris S McSweeney, Lindsay I Sly, Michael A McGuckin, Timothy H J Florin. 2010. “Mucolytic bacteria with increased prevalence in IBD mucosa augmentIn vitro utilization of mucin by other bacteria.” *Official journal of the American College of Gastroenterology | ACG* 105: 2420-2428. https://doi.org/10.1038/ajg.2010.281

**
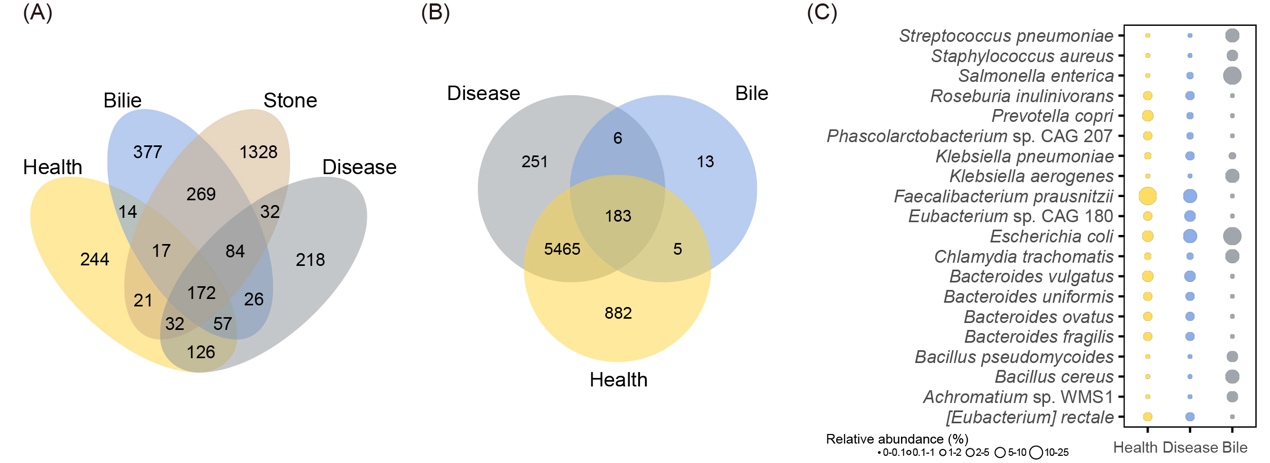
Figure S1** **Gut and biliary tract microbiome community**. (A−B) Venn diagram of the ASVs (A) and species (B) among groups. (C) Relative abundance of the top 20 bacterial communities in both groups at the level of species. ASVs, amplicon sequence variants.

**
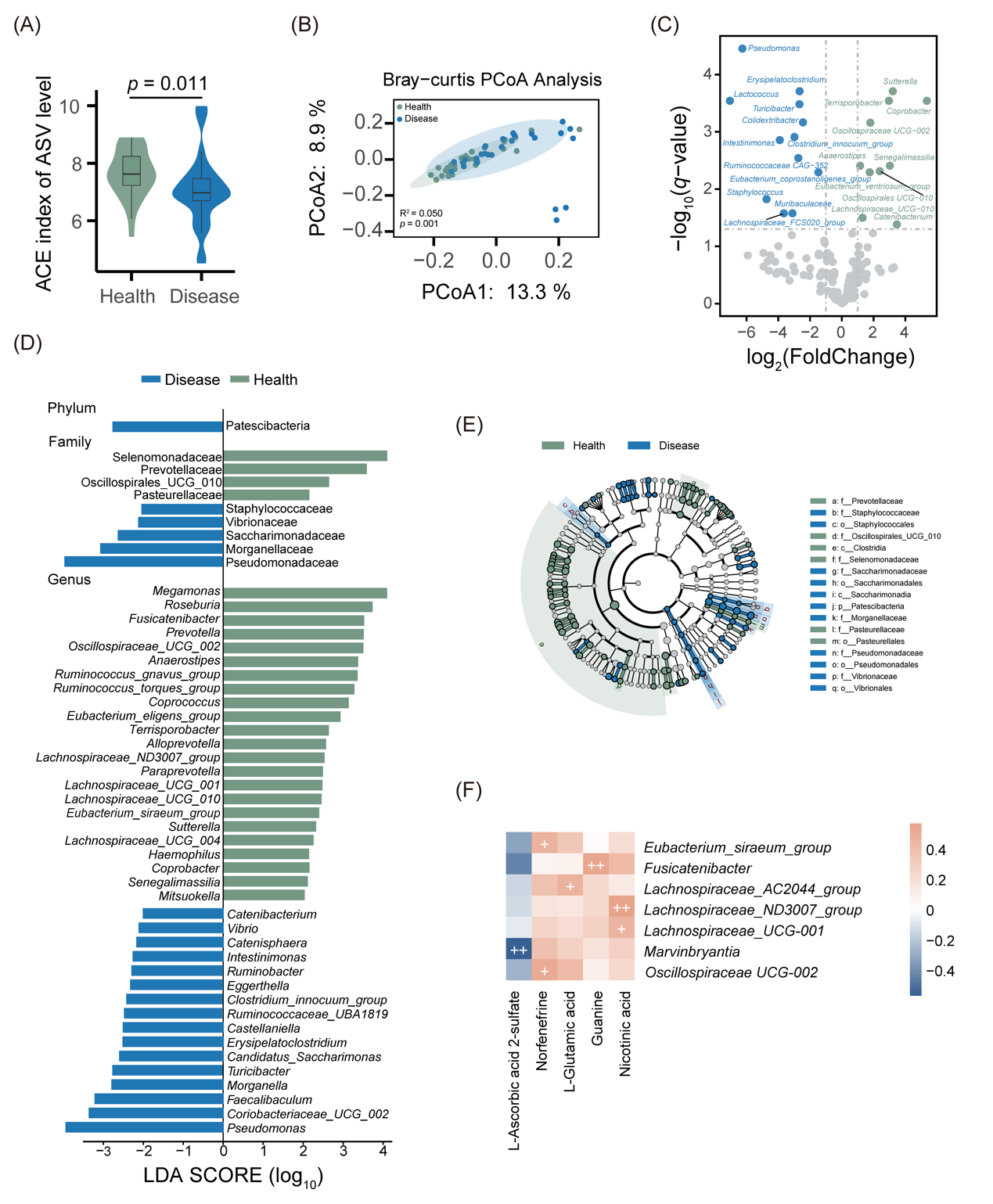
Figure S2** **The difference of gut microbiota in CGS (*n* = 30) and HCs (*n* = 30) according to the 16S rRNA data.** (A) The ACE index analysis between two groups at the level of ASVs. (B) Principal coordinate analysis (PCoA) of the microbiota based on the bray-curtis distance metrics for CGS and HCs. ANOSIM, R^2^ = 0.050, *p* = 0.001. (C) Volcano plot of the genus. Blue and green points represent the sample of those with *p* < 0.05 by Wilcoxon test (unadjusted *p*). The blue and green colors indicate a decrease and increase in abundance, respectively. (D) LDA scores for the bacterial taxa are differentially abundant between CGS and HCs (LDA > 3.5). Blue bars indicate taxa were enrichment in CGS, and green bars indicate taxa were enrichment in HCs. (E) Cladograms generated by LEfSe indicating differences in the bacterial taxa between CGS and HCs. Blue bars indicate taxa were enrichment in CGS, green bars indicate taxa were enrichment in HCs. (F) Spearman correlations between different metabolites and gut microbial genus. Significant correlations denoted by white signs (**^+^***p* < 0.1; **^++^***p* < 0.05, Student’s *t*-test (two-sided), Benjamini-Hochberg adjustment for multiple comparisons. Exact *p* values are provided in Table S5). CGS, cholesterol gallstone patients; HCs, healthy controls; ASVs, amplicon sequence variants.

**
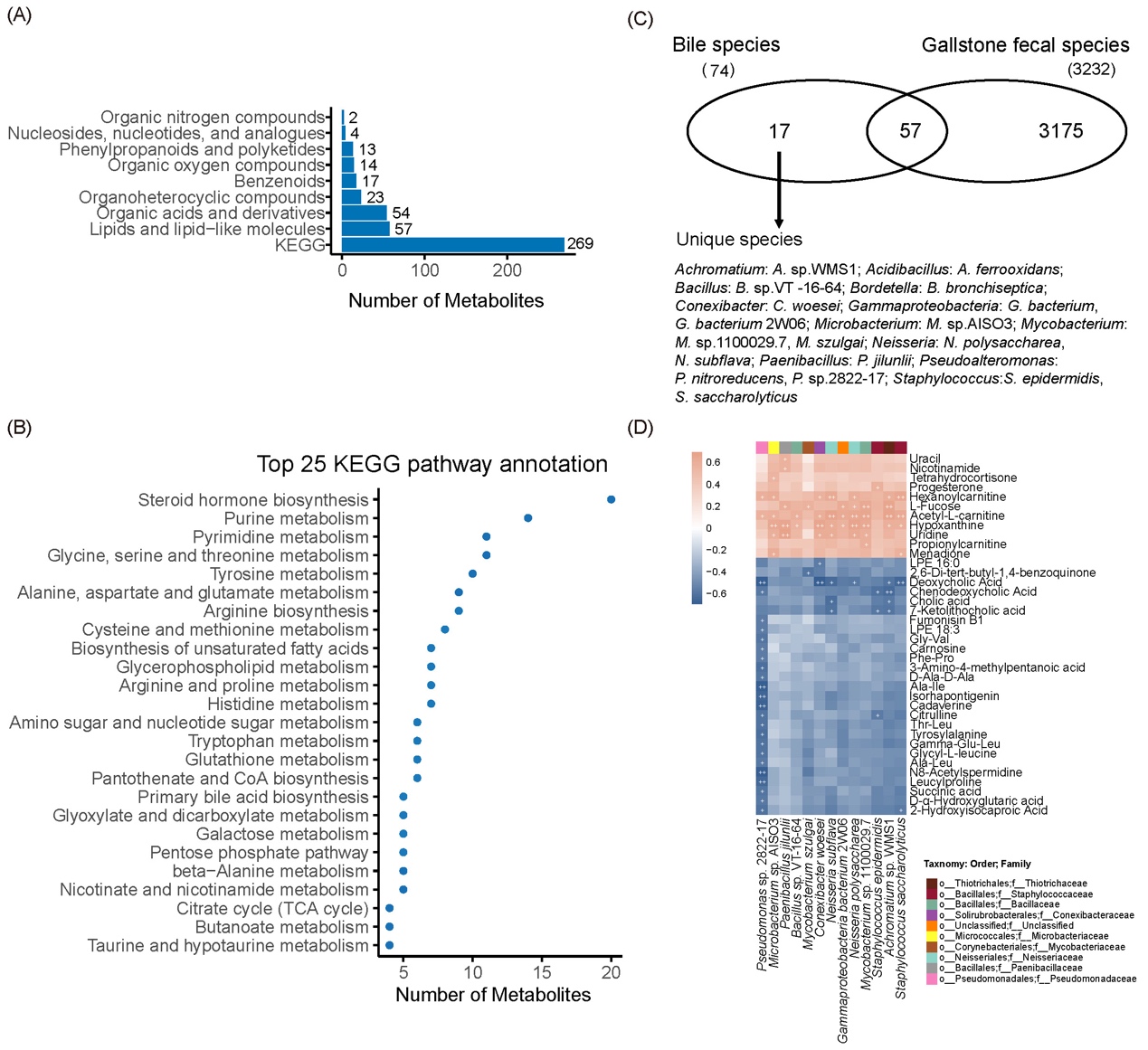
Figure S3 Conjoint analysis of microbiota and metabolites in bile samples.** (A) Metabolites were identified based on HMDB and KEGG. (B) The top 25 enrichment KEGG pathway. The x-axis represents the number of bile metabolites. (C) Unique microbial species in bile. (D) Spearman correlations between bile metabolites and bile unique microbial species. Significant correlations denoted by white signs (**^+^***p* < 0.1; **^++^***p* < 0.05, Student’s *t*-test (two-sided), Benjamini-Hochberg adjustment for multiple comparisons. Exact *p* values are provided in Table S14).

**
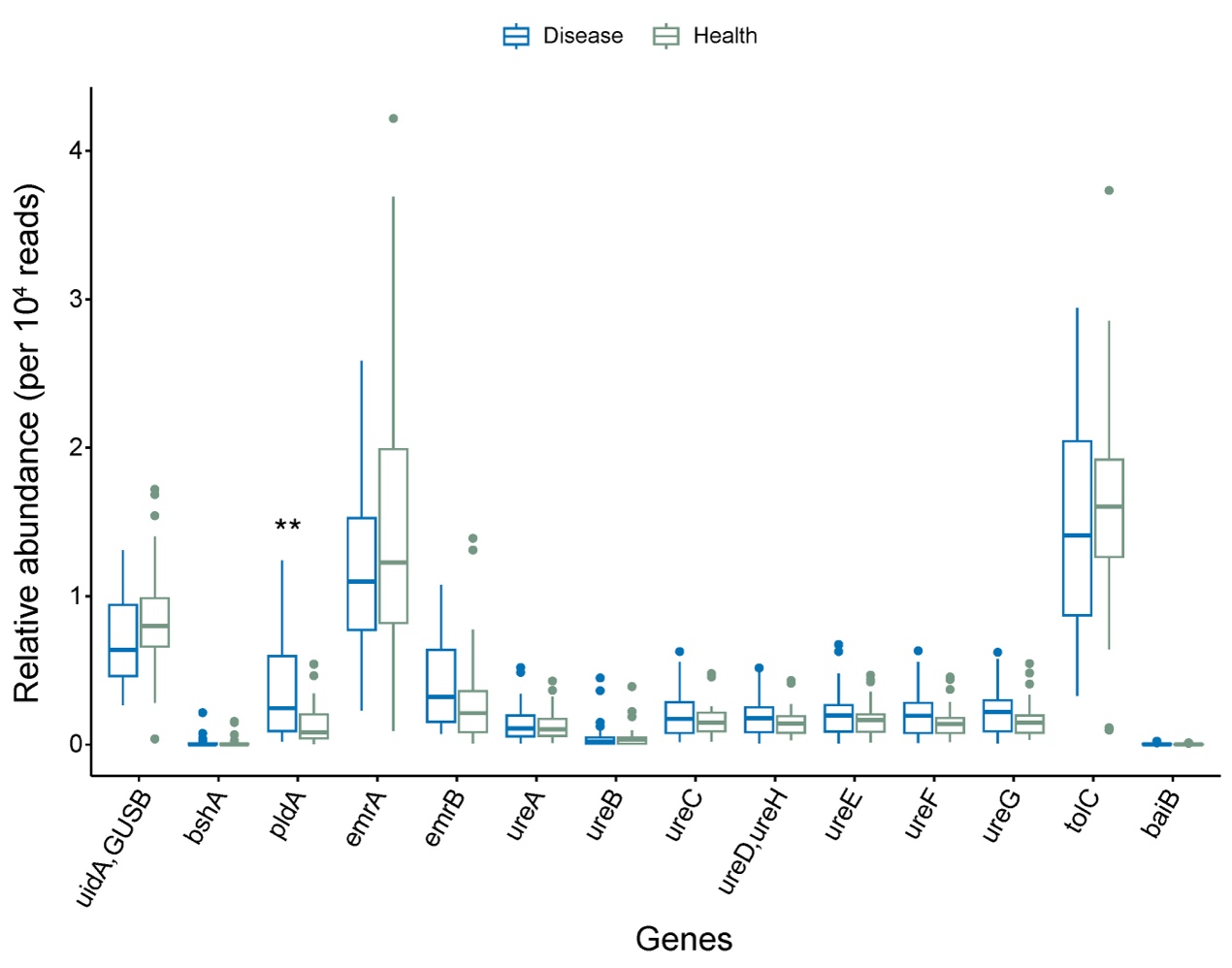
 Figure S4** Genes possibly associated with gallstones in previous studies.
